# Inter- and intra-examiner agreement of three classification systems of gingival recession

**DOI:** 10.15171/japid.2019.001

**Published:** 2019-08-31

**Authors:** Fatemeh Sarlati, Omid Moghaddas, Reza Shabahangfar, Sara Safari, Naser Valaei

**Affiliations:** ^1^Department of Periodontics, Dental Branch, Islamic Azad University, Tehran, Iran; ^2^Faculty Member, Thalassemia Research Center, Mazandaran University of Medical sciences, Sari, Iran; ^3^Faculty Member, Dental Branch, Islamic Azad University, Tehran, Iran

**Keywords:** Classification, gingival recession, periodontium

## Abstract

**Background:**

Several classifications have been proposed for gingival recession defects. Correct diagnosis of the type of gingival recession is necessary for proper treatment planning and assessment of the prognosis. Considering the existing uncertainty regarding the reliability of different classification systems available for gingival recession and their shortcomings, this study sought to assess the reproducibility and reliability of accuracy of three available classifications (Cairo, Mahajan and Miller’s classification systems) for gingival recession.

**Methods:**

This descriptive study was conducted on 32 patients presenting to the Department of Periodontics, who were selected using convenience sampling. The screening process entailed two sessions and those with a minimum of one site of gingival recession disclosing the cementoenamel junction (CEJ) of the tooth with no adjacent tooth loss at the site of recession were enrolled. Each patient was separately evaluated by three calibrated examiners twice with a minimum of one-week interval. Grading of the gingival recession defects was determined using the Cairo, Mahajan and Miller’s classification systems for gingival recession. The gradings of each examiner were separately recorded by a blinded examiner. A total of 120 single recession defects were examined and data were analyzed using intra-class correlation coefficient (ICC) and Spearman’s test. Level of agreement was evaluated according to Landis and Koch.

**Results:**

The results showed that the reliability of all the three methods was almost perfect (P<0.05), and no significant difference was noted in reliability of the Cairo, Mahajan and Miller’s classifications for gingival recession (P=0.7).

**Conclusion:**

Based on the results of the study, the highest intra- and inter-observer agreement in the use of the three classifications belonged to the Cairo classification; however, all the three classifications showed high reliability

## Introduction

Gingival recession is characterized by the apical migration of the gingival margin relative to the cementoenamel junction (CEJ).^1^ Gingival recession in patients with periodontitis mainly occurs at the interproximal areas; also it is often evident on the buccal surfaces of teeth in patients with good oral hygiene.^2,3^ The prevalence of gingival recession, involving one or multiple areas, has been reported to be 88% in patients >65 years of age and 50% in 18‒64-year-olds.^4^

Several classification systems have been introduced to classify gingival recession defects. Sullivan and Atkins^5^ in 1968 were the first to classify gingival recession of mandibular incisors into four groups of narrow, wide, deep and shallow according to the depth and width of recession defect and reported superior results for root coverage with the use of a free gingival graft for shallow and narrow defects. In another study, Mlinek et al^6^ characterized the shallow and narrow defects by the presence of less than 3 mm of gingival recession, and deep and wide defects by the presence of more than 3 mm of gingival recession. Later on, Miller presented a classification comprising of four groups of marginal gingival recession according to the position of the mucogingival junction and alveolar bone level.^7^ According to the Miller’s classification, recession of marginal gingiva, relative to the position of mucogingival junction and alveolar bone level, is divided into four classes as follows:

**Class I:** Recession of gingival margin not extending to the mucogingival junction, absence of bone loss and soft tissue loss in the interproximal region; the recession defect may be narrow or wide.

**Class II:** Recession of gingival margin extending to the mucogingival junction or beyond it, absence of bone loss and soft tissue loss in the interproximal region; the recession defect may be narrow or wide.

**Class III:** Recession of gingival margin extending to the mucogingival junction or beyond it, presence of bone loss or soft tissue loss at the interproximal area or presence of malposed teeth.

**Class IV:** Recession of gingival margin extending to the mucogingival junction or beyond it, presence of severe bone loss or soft tissue loss at the interproximal area or presence of severely malposed teeth.^7^ In 1997, Smith^8^ assessed the vertical and horizontal extension of gingival recession and classified horizontal defects using a 0-5-point scale (based on the severity of CEJ exposure), and vertical defects (measured by a probe) using a 0-9-point scale. Mahajan modified the Miller’s classification and suggested separating the two criteria of extension to the mucogingival junction and interproximal hard (bone) and soft tissue recession as follows:^9,10^

**Class I:** Gingival recession with no extension to the mucogingival junction.

**Class II:** Gingival recession extending to the mucogingival junction or beyond it.

**Class III:** Gingival recession with hard and soft tissue loss at the interproximal area exposing one-third of the root surface or presence of malposed teeth.

**Class IV:** Gingival recession with hard and soft tissue loss at the interproximal area exposing more than one-third of the root surface or presence of severely malposed teeth.

Accordingly, class III and class IV were different in terms of the severity of hard and soft tissue loss, and the prognosis of treatment based on this classification was considered the best for classes I and II with thick gingival biotype, good for classes I and II with thin gingival biotype, fair for class III with thick gingival biotype and poor for classes III and IV with thin gingival biotype. Rotundo et al^11^ introduced another classification system based on three factors: the amount of keratinized tissue, the presence/absence of non-carious cervical lesions and the presence/absence of interproximal attachment loss. Cairo et al^12^ suggested another classification in 2011 in terms of the attachment loss in the buccal and proximal areas using a measurement scale with three classes. The classification by Cairo is based on the attachment loss on the buccal and proximal surfaces and has three classes as follows:

**RT1:** Gingival recession with no loss of interproximal attachment. Interproximal CEJ was clinically not detectable at both mesial and distal aspects of the tooth.

**RT2:** Gingival recession associated with loss of interproximal attachment. The amount of interproximal attachment loss (measured from the interproximal CEJ to the depth of the pocket) was less than or equal to the buccal attachment loss (measured from the buccal CEJ to the depth of the buccal pocket).

**RT3:** Gingival recession associated with loss of interproximal attachment. The amount of interproximal attachment loss (measured from the interproximal CEJ to the depth of the pocket) was higher than the buccal attachment loss (measured from the buccal CEJ to the depth of the buccal pocket).

This study aimed to assess the reproducibility and reliability in accuracy of the three classification systems by Miller, Mahajan and Cairo as common subjective and objective methods in the evaluation of gingival recession.

## Methods

This study was carried out on patients presenting to the Department of Periodontics after approval by the ethic committee of the university. The patients were selected using convenience sampling and the study protocol was explained to them and informed consent was signed by each patient enrolled in this study.

All the selected patients had completed phase I periodontal therapy and were examined on their recall session. The inclusion criterion consisted of the presence of at least one site of single gingival recession disclosing the CEJ with no adjacent tooth loss. Also, teeth with crowns or restorations covering the CEJ and those with erosions or attrition at the site of CEJ were excluded. Sample size was calculated at 120 teeth for evaluation of inter- and intra-observer agreement according to the results of a pilot study on 20 patients, with a confidence interval of 1.5 (α=0.05) and study power of 90% (β=0.01).^13^

Clinical examinations were performed by three periodontists with different levels of clinical experience. The first (OM), second (MSH) and third (FS) examiners were coded A, B and C, respectively, to complete the datasheets blindly. The three periodontists were calibrated in a pilot study conducted on 20 patients. Clinical examination was performed on a dental chair in the Department of Periodontics using a periodontal probe (UNC15, CP, Hu-Friedy). Gingival phenotype and tooth shape (triangular, ovoid and square) were also recorded in the datasheets and a photograph was also taken of the site using a digital camera (PC1356; Canon, Tokyo, Japan). The photograph was taken using flash macro-photography with the Frankfurt plane parallel to the ground. Next, all the three periodontists examined the patients in terms of gingival recession using the three classifications by Miller, Mahajan and Cairo.

Each examiner separately examined each patient with no time limitation set. The results of the examinations by each examiner were recorded by a dental student in a datasheet. The examiners were blinded to the results provided by other examiners. The patients were then discharged and a recall session was scheduled at least one week later for re-examination (to assess intra-examiner agreement). The same process was repeated at the recall session and the results were recorded again by the same dental student. The results of both clinical examinations of each patient by each periodontist were separately collected and analyzed using ICC and Spearman’s test. Level of agreement was assessed according to Landis and Koch in six levels (Table 1).^14,15^

**Table 1:** Level of agreement according to Landis and Koch

**Poor agreement**	0.00
**Slight agreement**	0.00-0.20
**Fair agreement**	0.21-0.40
**Moderate agreement**	0.41-0.60
**Substantial agreement**	0.61-0.80
**Almost perfect agreement**	0.81-1.00

## Results

A total of 120 teeth in 32 patients, including 15 females and 17 males, with a mean age of 47.75±17.30 years were evaluated. Table 2 shows the frequency of different classes of gingival recession in the areas examined using the three classification systems. Table 3 shows the intra- and inter-observer agreements based on the examined site and evaluated parameters.

**Table 2:** Frequencies of different classes of gingival recession in the areas examined using the three classification systems

**Regions**		**Max.Ant**	**Man.Ant**	**Max.Post**	**Man.Post**	**Ant.Teeth**	**Post. Teeth**	**Max.Teeth**	**Man.Teeth**
**N**	120	7	94	4	15	101	19	11	109
**Miller's Classification (%)**									
**Class I**	3.3	1.1	1.4	0.4	0.4	2.6	0.7	1.5	0.1
**Class II**	13.3	0.7	11	0	1.7	11.7	1.7	0.7	12.6
**Class III**	65.3	3.75	50.7	2.9	7.9	54.4	1.8	6.7	58.6
**Class IV**	18.05	0.3	15.2	0	2.5	15.5	2.5	0.3	17.8
**Mahajan's Classification(%)**									
**Class I**	3	1.1	1.1	0.4	0.55	2.2	1	1.5	1.7
**Class II**	17.4	1.1	14.4	0.3	1.5	15.55	1.8	1.4	16
**Class III**	61	3	47	2.6	8.2	50	10.8	5.7	55.2
**Class IV**	18.6	0.3	15.9	0	1.2	16.25	2.3	0.4	18.2
**Cairo's Classification(%)**									
**Class I**	4.9	1.7	2.8	0	0.4	4.4	0.4	3.3	3.2
**Class II**	42.6	3.6	29.3	3.2	6.5	32.9	9.7	6.8	35.8
**Class III**	52.5	0.55	46.25	0.1	5.55	46.8	5.7	6.9	51.8
**Phenotype (%)**									
**Thinandhighscalloping**	81	5	62.6	2.8	10.5	67.6	13.3	7.8	73.2
**Thickandlowscalloping**	19	83	16.25	0.55	1.4	17.1	1.9	1.4	17.6
**ToothShape**									
**Long &narrow**	93.6	5.1	74.6	3.3	10.5	79.7	13.9	8.5	85.1
**Short&wide**	6.4	0.7	4.3	0	1.4	5	1.4	0.7	5.7

**Table 3:** Intra- and inter-observer agreements in terms of the evaluated sites

**Regions**	**All Regions**	**Max. Ant Teeth**	**Man.Ant Teeth**	**Max. Post Teeth**	**Man. Post Teeth**	**Ant Teeth**	**Post Teeth**	**Max. Teeth**	**Man Teeth**
n	120	7	94	4	15	101	19	11	109
**Miller's Classification(%)**									
Inter-observer agreement	0.64 (0.55;0.72)	0.47	0.66	0.83	0.57	0.65	0.70	0.60	0.65
Intra-observer agreement									
A	0.89 (0.83; 0.94)	0.57	0.90	0.75	1	0.88	0.94	0.63	0.91
B	0.86 (0.79; 0.92)	1	0.87	1	0.73	0.88	0.78	1	0.85
C	0.80 (0.72; 0.87)	0.85	0.83	0.50	0.66	0.83	0.63	0.72	0.80
**Mahajan's Classification (%)**									
Inter-observer agreement	0.58 (0.49; 0.66)	0.42	0.61	0.75	0.51	0.58	0.58	0.45	0.60
Intra-observer agreement									
A	0.87 (0.80; 0.93)	0.57	0.89	0.75	0.93	0.87	0.89	0.63	0.90
B	0.83 (0.76; 0.89)	1	0.80	0.50	1	0.82	0.89	0.82	0.83
C	0.75 (0.67; 0.82)	0.71	0.78	0.50	0.60	0.78	0.88	0.63	0.67
**Cairo's Classification(%)**									
Inter-observer agreement	0.68 (0.59; 0.76)	0.85	0.67	1	0.60	0.68	0.68	0.91	0.66
Intra-observer agreement									
A	0.95 (0.91; 0.98)	1	0.96	0.75	0.93	0.97	0.89	0.91	0.96
B	0.82 (0.75; 0.88)	0.85	0.82	1	0.80	0.82	0.84	0.91	0.81
C	87 (0.80; 0.93)	1	0.85	1	0.93	0.86	0.94	1	0.86
**Phenotype (%)**									
Inter-observer agreement	0.92 (0.87;0.96)	0.81	0.93	0.83	0.91	0.92	0.89	0.82	0.93
Intra-observer agreement									
A	0.96 (0.92; 0.99)	1	0.94	1	1	0.95	1	1	0.95
B	0.96 (0.92; 0.99)	1	0.94	1	1	0.95	1	1	0.95
C	0.96 (0.92; 0.99)	1	0.94	1	1	0.95	1	1	0.95
**Tooth shape**									
Inter-observer agreement	0.95 (0.91; 0.98)	0.81	0.97	1	0.91	0.96	0.93	0.88	0.96
Intra-observer agreement									
A	1	1	1	1	1	1	1	1	1
B	1	1	1	1	1	1	1	1	1
C	1	1	1	1	1	1	1	1	1

The highest intra-observer agreement in use of the Miller’s classification belonged to the anterior teeth. The level of agreement was slightly higher for mandibular anterior teeth, which was almost perfect. The highest inter-observer agreement was noted in the maxillary posterior teeth with almost perfect agreement (Table 3).

The highest intra-observer agreement in the classification by Cairo belonged to the maxillary teeth, which was slightly higher in the anterior teeth. The agreement in this region was almost perfect. The highest inter-observer agreement was noted in the maxillary posterior teeth with almost perfect level of agreement (Table 3). The highest rate of inter- and intra-observer agreement among the three classifications by Miller, Mahajan and Cairo belonged to Cairo; however, all three showed high reliability in terms of intra-observer agreement, with almost perfect level of agreement. In terms of inter-observer agreement, Cairo and Miller’s classifications showed substantial level of agreement, and the Cairo classification showed slightly higher reliability than the Miller’s classification. The inter-observer agreement was moderate for Mahajan’s classification (Table 3).

The highest intra-observer agreement was noted in the assessment of the gingival phenotype of the maxilla. The level of agreement for all the areas was found to be almost perfect. The highest inter-observer agreement was noted in the mandibular teeth, which was slightly higher in the mandibular anterior teeth. However, the agreement was almost perfect in all the areas (Table 3).

In the assessment of the shape of teeth, almost perfect inter- and intra-observer agreement was seen in all the areas (Table 3).

The reliability of the Cairo’s classification was higher than that of Miller’s and the reliability of Miller’s classification was higher than that of Mahajan’s. In other words, the intra-examiner reliability in the two examinations by use of the Cairo’s classification was higher than that with the use of Miller’s classification and the latter was higher than that with the use of Mahajan’s. The inter-observer reliability among the three periodontists with the use of Cairo’s classification was higher than that with the use of Miller’s classification and the latter was higher than that of Mahajan’s (Tables 4 and 5).

**Table 4:** Intra-observer reliability in the use of each of the three classification systems

**Methods**		**Miller**				**Mahajan**				**Cairo**		
**Differences**		**0**	**1**	**2**	**3**	**0**	**1**	**2**	**3**	**0**	**1**	**2**
**Observers**	**A**	107(89.2%)	11 (9.2%)	2 (1.7%)	0	105 (87.5%)	13 (10.8%)	2 (1.7%)	0	112 (93.3%)	8 (6.7%)	0
	**B**	104 (86.7%)	16 (13.3%)	0	0	100 (83.3%)	20 (16.7%)	0	0	99 (82.5%)	21 (17.5%)	0
	**C**	96 (80%)	18 (15%)	6 (5%)	0	90 (75%)	25 (20.8%)	5 (4.2%)	0	105 (87.5%)	15 (12.5%)	**0**
**Total**		307 (85.3%)	45 (12.7%)	8 (2%)	0	295 (81.9%)	58 (16.1%)	7 (2%)	0	316 (87.8%)	44 (12.2%)	**0**

**Table 5:** Inter- and intra-observer reliability in the use of each of the three classification systems

**Methods**		**Miller**				**Mahajan**				**Cairo**			
**Differences**		**0**	**1**	**2**	**3**	**0**	**1**	**2**	**3**	**0**	**1**	**2**
**Observers**	**B&A**	80 (66.7%)	32 (26.7%)	8 (6.7%)	**0**	76 (63.3%)	34 (28.3%)	10 (8.3%)	**0**	80 (66.7%)	37 (30.8%)	3 (2.5%)
	**C&A**	67 (55.8%)	46 (38.3%)	7 (5.8%)	**0**	64 (53.3%)	48 (40%)	8 (6.7%)	**0**	84 (70%)	32 (26.7%)	4 (3.3%)
	**C&B**	86 (71.7%)	29 (24.2%)	5 (4.2% 0**)**	**0**	71 (59.7%)	48 (40%)	1 (0.8%)	**0**	83 (69.2%)	35 (29.2%)	2 (1.7%)
**Total**		233 (64.7%)	107 (29.7% 0)	20 (5.6%)	**0**	211 (58.6%)	130 (36.1%)	19 (5.3%)	**0**	247 (68.6%)	104 (28.9%)	9 (2.5%)

Table 6 shows the level of agreement for all the three classifications. According to the correlation coefficients calculated, all the three methods had significantly high reliability (almost perfect; P<0.05) and no significant difference was found in reliability between the three classification systems (P=0.7).

**Table 6:** Level of agreement in the use of the three classification systems for gingival recession

**Agreement level** **Classification**	**Poor agreement 0.00**	**Slight agreement 0.00 – 0.20**	**Fair agreement 0.21 – 0.40**	**Moderate agreement 0.41 – 060**	**Substantial agreement 0.61 – 0.80**	**Almost perfect agreement 0.81 – 1.00**
**Miller**	--	--	--	8 %2	45 (%12.7)	307 (%85.3)
**Mahajan**	--	--	--	7 %2	58 (%16.1)	295 (%81.9)
**Cairo**	--	--	--	--	44 (%12.2)	316 (%87.8)

## Discussion

The results of this study showed that the highest inter- and intra-observer agreement among the three classification systems by Cairo et al, Mahajan and Miller belonged to the Cairo’s classification. However, all the three classification systems showed high level of intra-observer agreement (almost perfect). Cairo et al^12^ in 2011 evaluated the inter- and intra-observer agreement and reported almost perfect intra- and inter-observer agreement, which was in line with our results. The similarity of the results of the two studies is probably due to the fact that both studies evaluated teeth with gingival recession and detectable and visible CEJ. Moreover, since assessment of the position of interproximal papilla is important in correct classification of gingival recession in all the systems, we excluded teeth with tooth loss adjacent to the site of recession (in addition to the afore-mentioned exclusion criteria) in order to eliminate the effect of physiological gingival recession on the results, which occurs adjacent to an edentulous area.

Mahajan et al^16^ used a modified version of the Miller’s classification suggested by the same authors in previous publication in order to compensate for the shortcomings of Miller’s classification and obtain more reliable results. They assessed the inter- and intra-observer agreement for determination of the class of gingival recession using Mahajan’s classification and reported almost perfect inter- and intra-observer agreement, which was in accordance with our results. However, we assessed the reliability of three classification systems in our study and did not find any superiority for the Mahajan’s classification over that of Miller’s as reported by Mahajan et al.^10^ In our study, the reliability of the Miller’s classification was slightly higher than that of Mahajan.

Our data is also in agreement with the findings of Bertl et al^17^ in 2015 on the inter- and intra-observer agreement for several parameters, revealing substantial to almost perfect intra-observer agreement in the use of Miller’s classification, (in our study, level of inter-observer agreement was almost perfect). In the study by Bertl et al gingival phenotype and tooth shape were evaluated. The level of inter- and intra-observer agreement in the assessment of gingival biotype was slight to moderate. This level was fair to moderate for tooth shape. However, in our study, level of inter- and intra-observer agreement was found to be almost perfect. The difference between our results and those of Bertl et al might be explained by the following:

They evaluated the inter- and intra-observer agreement in identification of the CEJ. They showed that the inter-observer agreement was slight to fair and the intra-observer agreement was poor to almost perfect. The levels of agreement for identification of CEJ (which would affect all the other parameters in their study) were very low. Also, the range of agreement was very wide. These factors affect tooth shape and use of Miller’s classification. Since Bertl et al reported that inability to identify CEJ was a limitation of their study (due to factors such as restorations at the CEJ), we excluded cases with undetectable CEJ and wear or restoration at this area. Identification of CEJ plays a significant role in increasing the level of agreement among the examiners. On the other hand, Bertl et al evaluated photographs obtained from teeth while in our study, the examiners examined actual patients twice with a one-week interval using UNC15 probe, which definitely explains the reason for obtaining more accurate results, especially at the inter-proximal areas. However, Bertl et al evaluated equal number of teeth (n=50) from both the anterior and posterior regions of the mandible and maxilla while defects evaluated in our study were mostly located in the anterior mandible.

Another study used a new classification based on the presence of three factors namely the amount of keratinized gingiva, presence/absence of non-carious cervical lesions and presence/absence of proximal attachment loss.^11^ They showed almost perfect intra-observer agreement, which was in line with our results; however, the inter-observer agreement was moderate, which was lower than the level of agreement reported in our study. Although parameters evaluated in their study were slightly different from ours, assessments were made on factors that have a direct effect on agreement with the use of different classification systems for gingival recession (which were evaluated in our study). One limitation of their study and the classification system offered was simultaneous evaluation of non-carious cervical lesions and inter-proximal attachment loss since these lesions can complicate correct detection of CEJ. Another limitation was small sample size (10 patients; three females and seven males), which affects the results. Thus, we evaluated 32 patients, including 17 males and 15 females that is a small representative of actual population of patients.

## Conclusion

Our results did not reveal a significant difference in the reliability of the three classification systems for gingival recession. The reliability of all the three systems was high and each of them can be used for assessment and classification of gingival recession in patients. The noteworthy issue is to precisely identify the CEJ and determine the proximal attachment loss and other parameters affecting the results.
